# Injuries and Training Practices in Competitive Adolescent Distance Runners: A Retrospective Cross-Sectional Study

**DOI:** 10.3389/fspor.2021.664632

**Published:** 2021-06-24

**Authors:** Robert H. Mann, Carly D. McKay, Bryan C. Clift, Craig A. Williams, Alan R. Barker

**Affiliations:** ^1^Children's Health and Exercise Research Centre, Sport and Health Sciences, University of Exeter, Exeter, United Kingdom; ^2^Centre for Motivation and Health Behaviour Change, Department for Health, University of Bath, Bath, United Kingdom; ^3^Centre for Qualitative Research, University of Bath, Bath, United Kingdom; ^4^Department for Health, University of Bath, Bath, United Kingdom

**Keywords:** youth, endurance, health, epidemiology, track and field, performance, athlete health protection, injury prevention

## Abstract

**Background:** Distance running is one of the most popular sports around the world. The epidemiology of running-related injury (RRI) has been investigated in adults, but few studies have focused on adolescent distance runners.

**Objectives:** (1) To provide descriptive epidemiology of RRI (risks, rates, body regions/areas, and severity) and examine the training practices (frequency, volume, and intensity) of competitive adolescent distance runners (13–18 years) in England, and (2) to describe potential risk factors of RRI.

**Methods:** A cross-sectional study design was used. Adolescent distance runners (*n* = 113) were recruited from England Athletics affiliated clubs. Participants voluntarily completed an online questionnaire between April and December 2018. At the time of completion, responses were based on the participant's previous 12-months of distance running participation. Incidence proportions (IP) and incidence rates (IR) were calculated.

**Results:** The IP for “all RRI” was 68% (95% CI: 60–77), while the IR was 6.3/1,000 participation hours (95% CI: 5.3–7.4). The most commonly injured body areas were the knee, foot/toes, and lower leg; primarily caused by overuse. The number of training sessions per week (i.e., frequency) significantly increased with chronological age, while a large proportion of participants (58%) self-reported a high level of specialisation.

**Conclusions:** RRI is common in competitive adolescent distance runners. These descriptive data provide guidance for the development of RRI prevention measures. However, analytical epidemiology is required to provide better insight into potential RRI risk factors in this specific population.

## Introduction

Distance running is one of the most popular sports around the world (Hulteen et al., 2017). Although associated with numerous health benefits (Pedisic et al., 2019), distance running is also associated with adverse health outcomes, such as running-related injury (RRI) (van Gent et al., 2007; Videbaek et al., 2015). RRIs are typically located in the lower extremity, with the lower leg, knee, and foot/toes being the most commonly injured body areas (van Gent et al., 2007). Systematic reviews highlight that RRI rates range from 2.5 to 33.0 injuries per 1,000 h of participation in adult distance runners (Videbaek et al., 2015), whereas incidence proportions range from 3.2 to 79.3% (van Gent et al., 2007; Kluitenberg et al., 2015). This variation may be explained by differences in research methodology (Tabben et al., 2019). Regardless, there is much less research that has exclusively examined RRIs in adolescent (13-18 years) distance runners (Rauh et al., 2000, 2006; von Rosen et al., 2017; Mann et al., 2021). For example, the majority of previous studies have included all track and field (athletics) disciplines, including distance runners within sub-sample analyses (Jacobsson et al., 2013; Pierpoint et al., 2016; von Rosen et al., 2018; Carragher et al., 2019; Martínez-Silván et al., 2020). Given that distance running is the second most prevalent sport amongst adolescents in England (Sport England., 2021), this lack of research is concerning. As a result, a better understanding of common RRIs during maturation is important for supporting long-term athletic development (Bergeron et al., 2015; Krabak et al., 2020).

Although excelling as an adolescent track and field athlete is unessential for success as a senior athlete (Moesch et al., 2011; Kearney and Hayes, 2018), research has shown that 90% of youth athletes in the United Kingdom decide to specialise in a chosen discipline from an early age (13–14 years) (Shibli and Barrett, 2011). Research has also indicated that youth sport specialisation is positively associated with injury history (Fabricant et al., 2016; Post et al., 2017a). Therefore, the trend towards early sport specialisation is an issue in relation to adolescent distance runners (Myer et al., 2015), whereby “success” for endurance athletes is often attributed to consistent and monotonous training intensities, durations, and frequencies (Seiler, 2010). In turn, it is feasible that certain training practices may increase the risk of sustaining an RRI (Nielsen et al., 2012). Yet, little is known about the training practices of adolescent distance runners. This is largely the case for whether issues related to age, growth, and maturation contribute to RRI in this population too (Wik et al., 2020). As a result, the primary purpose of this study was to provide descriptive epidemiology of RRI (i.e., risks, rates, body regions/areas, and severity) and examine the training practices (i.e., frequency, volume, and intensity) of competitive adolescent distance runners in England, in relation to sex and age-group differences. The secondary purpose was to describe potential risk factors (correlates) of RRI in this population.

## Methods

### Study Design

This was a cross-sectional study based on the completion of an online questionnaire. Data collection took place between April and December 2018. Ethics approval was granted by the institutional ethics committee (171206/B/02). Based on findings from prior research (van Gent et al., 2007), an *a priori* target sample size of 151 participants was calculated. This sample size was based on having an ability to perform logistic regression analysis to identify an odds ratio of 1.75 between potential risk factors and RRI with an 80% power and alpha of 0.05.

### Participants

Participants were included if they were a member of an England Athletics affiliated club, aged between 13 and 18 years, and training for and/or competing in a distance running discipline (800 m up to 10,000 m, including steeplechase). Participants were excluded if they were unable to fully understand the study procedures, did not meet the inclusion criteria, and/or failed to complete the full questionnaire [i.e., one or more section(s) had not been completed]. Using convenience sampling, participants were recruited directly from England Athletics affiliated clubs, with study information being distributed via face-to-face meetings, email, and/or social media. Because of the nature of participant recruitment, it was not possible to determine the total number of athletes approached to take part in this study. Written parental consent and participant assent were obtained before completion of the questionnaire.

### Data Collection Procedure

#### Study Questionnaire

Participants completed the questionnaire via an online platform (Qualtrics XM., Provo, Utah, USA), which is compatible with computers and mobile devices. The questionnaire included sections related to: (1) background demographics (e.g., date of birth), (2) performance history (e.g., discipline preferences), (3) training practices (e.g., weekly training volume), (4) athletic identity (i.e., the degree to which an individual identifies with the status of being an athlete) (Brewer et al., 1993), (5) injury and medical history (e.g., RRIs and “pains or discomforts” during the previous 12-months), and (6) level of specialisation (i.e., the degree to which an individual is engaged in intense, year-round, and sport-specific training) (Jayanthi et al., 2013, 2015). Development of the questionnaire was based on methods employed in previous studies (Jacobsson et al., 2012; Huxley et al., 2014; Woollings et al., 2015). Key stakeholders were also involved in developing this questionnaire to ensure it was suitable for the target audience. This included adolescent distance runners, parents, coaches, and physiotherapists (*n* = 12). A copy of the questionnaire has been uploaded as a Supplementary Material.

#### Injury Definition and Classification

The primary outcome measure was RRI, defined as any physical complaint that resulted from distance running participation (training or competition), irrespective of the need for medical attention or time loss from distance running activities. This definition of RRI was named “all RRI” and included self-reported pains or discomforts. RRI needed to have occurred during the 12-months preceding questionnaire completion. This RRI definition aligns to the most relevant (and recent) consensus statements on injury and illness data collection processes (Timpka et al., 2014; Bahr et al., 2020). All RRIs and pains or discomforts were classified according to body region and area, aligned to the terminology applied in the track and field consensus statement (Timpka et al., 2014). RRIs requiring medical attention and/or resulted in time loss from full distance running participation were also recorded. Medical attention injuries were classified as any RRI that involved the assessment of a participant's condition by a medical or healthcare practitioner. Time loss injuries were classified according to their severity: none (0 days), slight (1 day), minimal (2–3 days), mild (4–7 days), moderately serious (8–28 days), serious (>28 days-6 months) or long-term (>6 months) (Timpka et al., 2014). Both broad and narrow definitions were used to capture the full range of RRIs (Clarsen and Bahr, 2014). When self-reporting an RRI (excluding pain or discomforts), participants were able to specify whether they had previously experienced this RRI (i.e., affecting the same body region/area). The first-author reviewed this information and classified the RRI as either being an index RRI or a recurrent RRI (Hamilton et al., 2011). Recurrent RRIs were counted as a separate RRI. Due to the study design, it was not possible to determine whether a recurrent RRI was either a reinjury or an exacerbation. Although mode of onset (gradual/sudden) and cause (traumatic/overuse) of RRI were self-reported by participants, this excluded any self-reported pains or discomforts, as they were not classified according to these outcomes.

#### Potential Intrinsic and Extrinsic RRI Risk Factors

Data collection included the assessment of potential intrinsic and extrinsic RRI risk factors. These risk factors were selected based on previous studies on a range of youth sports (Emery, 2003; Caine et al., 2008; Fabricant et al., 2016) and adult distance runners (van Gent et al., 2007). Potential intrinsic risk factors included sex, age-group, stature, body mass, body mass index (BMI), maturity timing and tempo, training age, performance level, level of specialisation, and history of previous injury. Potential extrinsic risk factors included discipline preferences, training practices (volume/intensity), use of a coach, inclusion of a warm-up/cool-down, and inclusion of strength and conditioning as part of their weekly training.

Age-group was categorised according to those age ranges applied in English Schools' Athletic Association competitions: 13–14 years (U15), 15–16 years (U17), and 17–18 years (U19). BMI was divided into three subgroups by applying age and sex specific cut points (i.e., underweight, normal, and overweight/obese) (Cole et al., 2000, 2007). To estimate maturity timing and tempo, each participant's age at peak height velocity (PHV) was determined by applying sex-specific maturity offset equations (Moore et al., 2015). Subsequently, maturity timing and tempo were categorised according to previous research (Baxter-Jones et al., 2005). Training age (i.e., number of years participating in distance running) and current performance level (i.e., club, country, regional, national, international) were self-reported. Level of specialisation was categorised as *low, moderate*, or *high* according to how participants responded to the following three questions: (1) Is distance running your main sport?, (2) Have you quit other sports in order to focus on distance running?, and (3) How many months of the past year did you participate in distance running? (Jayanthi et al., 2013, 2015). History of previous injury was self-reported by participants, defined as any RRI that preceded the 12-month retrospective period of data collection.

To establish discipline preferences, participants self-reported the distance running discipline that they most often trained for and/or competed in (i.e., primary discipline). Participants were also able to stipulate the total number of disciplines that they trained for and/or competed in. Selecting one discipline was categorised as a “single discipline preference,” while two or more disciplines was categorised as a “multi discipline preference.” Training practices were self-reported as hours per week and days per week. These variables were also used to calculate incidence rate (IR). To calculate internal training load (ITL), each participant self-reported their most recent training week, including the duration (minutes), distance (km), session type, and rating of perceived exertion (RPE) for each of their training sessions. Weekly session RPE (sRPE) was subsequently calculated, having been validated as a measure of ITL in adolescent distance runners (Mann et al., 2019). Use of a coach, inclusion of a warm-up/cool-down, and inclusion of a strength and conditioning programme were categorised via binary (i.e., yes/no) self-reported responses.

### Statistical Methods

The statistical software SPSS (version 26.0; IBM., Chicago, USA) and an Excel spreadsheet (Microsoft., Redmond, USA) were used to conduct all analyses. All completed questionnaires that met the inclusion criteria were reviewed by the first-author. Duplicate entries were excluded. This review process also enabled the first-author to check for obvious data entry errors (i.e., date of birth not aligning with age-group). No duplicates or data entry errors were identified by the first-author.

Incidence proportion (IP), reported as a percentage (%), was calculated to determine the average probability that a participant would sustain at least one RRI during the 12-month study period. Clinical incidence, reported per 100 participants/year, was calculated to determine the proportion of participants who experienced RRI during the 12-month study period. IR was calculated to establish the number of RRI per participant/1,000 h of exposure to distance running. To compare the relative risk between male and female participants, risk ratios (related to IP) and rate ratios (related to IR) were calculated. When reported, male participants were used as the referent group, meaning that the higher or lower risk/rate is related to the male participants (Knowles et al., 2006). 95% intervals (CI) were calculated for IP and IR (Marshall, 2005), in addition to the risk and rate ratios (Greenland and Rothman, 2008). Body regions and areas of RRI were described using frequencies and proportions (%). Median and interquartile range (IQR) statistics were calculated for multiple RRI and time loss RRI. Means and standard deviations (SD) were calculated for continuous variables. Percentages (%) were calculated for categorical variables. Sex differences were analysed using independent samples *t*-tests for continuous variables and Chi-squared tests (X^2^) for categorical variables. Differences between age-group training practices were analysed using one-way ANOVA. Pairwise comparisons were subsequently used to explore differences between age-groups. Statistical significance was set at an alpha level of 0.05, and effect sizes for mean comparisons were described using Cohen's thresholds (*small* = 0.2, *medium* = 0.5, *large* = 0.8) (Cohen, 1992).

## Results

### Descriptive Characteristics

A total of 115 adolescent distance runners (65 female) volunteered to participate in this study and completed the questionnaire. However, two participants were excluded from the results as they were not members of an England Athletics affiliated club, resulting in a sample of 113 adolescent distance runners (64 female). As the *a priori* target sample size was not achieved (see section Study Design), no logistic regression analysis was conducted. Participant descriptive characteristics, including a number of intrinsic and extrinsic risk factors, are shown in Table 1 with sex differences reported.

**Table 1:** Descriptive characteristics of the participants (data presented as mean and SD, unless otherwise stated).

**Characteristic**	**Overall (*n* = 113)**	**Male (*n* = 49)**	**Female (*n* = 64)**	***p*-value**	**Effect size**
Age, years	15.9 (1.5)	15.9 (1.5)	16.0 (1.6)	0.65	0.06
Training age, years	5.6 (2.4)	5.6 (2.1)	5.7 (2.5)	0.77	0.04
Age-group (*N*, %):				X^2^ = 0.94	
13–14 years	36 (32%)	16 (33%)	20 (31%)		
15–16 years	43 (38%)	19 (39%)	24 (38%)		
17–18 years	34 (30%)	14 (29%)	20 (31%)		
Stature, cm	168.8 (8.6)	173.7 (8.3)	165.0 (6.9)	<0.01	1.15
Body mass, kg	52.8 (8.7)	57.4 (9.4)	49.3 (6.0)	<0.01	1.06
BMI cut points (N, %):				X^2^ = 0.02	
Underweight	32 (28%)	8 (16%)	24 (38%)		
Normal	80 (71%)	40 (82%)	40 (62%)		
Overweight	1 (1%)	1 (2%)	0 (0%)		
Maturity timing (*N*, %):				X^2^ <0.01	
Pre-PHV	0 (0%)	0 (0%)	0 (0%)		
At-PHV	15 (13%)	12 (24%)	3 (5%)		
Post-PHV	98 (87%)	37 (76%)	61 (95%)		
Maturity tempo (*N*, %):				X^2^ = 0.11	
Early	0 (0%)	0 (0%)	0 (0%)		
Average	102 (90%)	47 (96%)	55 (86%)		
Late	11 (10%)	2 (4%)	9 (14%)		
Current performance level (*N*, %):				X^2^ = 0.62	
Club	23 (20%)	11 (22%)	12 (19%)		
County	25 (22%)	8 (16%)	17 (27%)		
Regional	19 (17%)	10 (20%)	9 (14%)		
National	41 (36%)	17 (35%)	24 (37%)		
International	5 (4%)	3 (6%)	2 (3%)		
Primary discipline (*N*, %):				X^2^ = 0.18	
800 m	39 (35%)	17 (35%)	22 (34%)		
1,500 m	34 (30%)	13 (27%)	21 (33%)		
3,000 m	14 (12%)	5 (10%)	9 (14%)		
5,000 m	11 (10%)	9 (18%)	2 (3%)		
10,000 m	1 (1%)	0 (0%)	1 (2%)		
Steeplechase	7 (6%)	3 (6%)	4 (6%)		
Cross-country	7 (6%)	2 (4%)	5 (8%)		
Total number of disciplines (*N*, %):				X^2^ = 0.46	
One (single)	8 (7%)	2 (4%)	6 (9%)		
Two or more (multiple)	105 (93%)	47 (96%)	58 (91%)		
Level of specialisation (*N*, %):				X^2^ = 0.61	
Low	9 (8%)	5 (10%)	4 (6%)		
Moderate	39 (35%)	15 (31%)	24 (38%)		
High	65 (58%)	29 (59%)	36 (56%)		
Training practices:					
Sessions per week	4.7 (1.5)	4.9 (1.6)	4.6 (1.5)	0.28	0.20
Weeks per month	4.0 (0.2)	4.0 (0.1)	3.9 (0.3)	0.15	0.27
Months per year	11.0 (2.1)	11.2 (1.7)	10.8 (2.3)	0.31	0.19
Average session duration (min)	57.6 (19.6)	56.1 (17.2)	58.8 (21.3)	0.46	0.14
Average RPE per session (AU)	6.4 (1.4)	6.2 (1.3)	6.5 (1.5)	0.25	0.21
Average sRPE per session (AU)	391.9 (177.7)	373.7 (158.7)	405.9 (191.1)	0.34	0.18
Have a coach (*N*, %):				X^2^ = 0.63	
Yes	109 (96%)	48 (98%)	61 (95%)		
No	4 (4%)	1 (2%)	3 (5%)		
Inclusion of a warm-up (*N*, %):				X^2^ = 0.43	
Yes	112 (99%)	48 (98%)	64 (100%)		
No	1 (1%)	1 (2%)	0 (0%)		
Inclusion of a cool-down (*N*, %):				X^2^ = 0.19	
Yes	111 (98%)	47 (96%)	64 (100%)		
No	2 (2%)	2 (4%)	0 (0%)		
Inclusion of a strength and conditioning programme (*N*, %):				X^2^ = 0.11	
Yes	89 (79%)	35 (71%)	54 (84%)		
No	24 (21%)	14 (29%)	10 (16%)		

*N, number; cm, centimetres; kg, kilograms; BMI, Body Mass Index; PHV, Peak Height Velocity; min, minutes; RPE, Rating of Perceived Exertion; sRPE, Session Rating of Perceived Exertion; AU, Arbitrary Units. NB, Due to rounding, not all numbers add up correctly*.

### Incidence Proportion and Incidence Rate

#### All RRI

Seventy-seven (68%) participants sustained at least one new RRI during the 12-month study period, including self-reported pains or discomforts. Thirty-six (47%) reported multiple RRI (median: 2; IQR: 1). The total number of RRI sustained was 142, resulting in an IP of 68% (95% CI: 60–77), and a clinical incidence of 126/100 participants/year (95% CI: 113–138). The overall IR was 6.3/1,000 participation hours (95% CI: 5.3–7.4). Eleven (10%) participants had a history of previous RRI.

Thirty-one (63%) male participants sustained 60 RRI [IP = 63% (95% CI: 50–77); clinical incidence = 122/100 participants/year (95% CI: 104–141)] compared with 46 (72%) female participants who sustained 82 RRI [IP = 72% (95% CI: 61–83); clinical incidence = 128/100 participants/year (95% CI: 111 to 145)], resulting in a risk ratio of 0.88 (95% CI: 0.68–1.14). The IR for male participants was 5.9/1,000 participation hours (95% CI: 4.6–7.6), and for female participants it was 6.6/1,000 participation hours (95% CI: 5.3–8.2), resulting in a rate ratio of 0.89 (95% CI: 0.68–1.33).

#### Time Loss RRI

Sixty-five (58%) participants sustained at least one new time loss RRI during the 12-month study period, including self-reported pains or discomforts. A total of 112 (79%) RRI resulted in at least one day of time loss from distance running, resulting in an IP of 58% (95% CI: 48–67), and a clinical incidence of 99/100 participants/year (95% CI: 97–101). The IR was 5.0/1,000 participation hours (95% CI: 4.1–6.0). The median amount of time loss was 7 days (IQR: 34).

**Figure 1:**
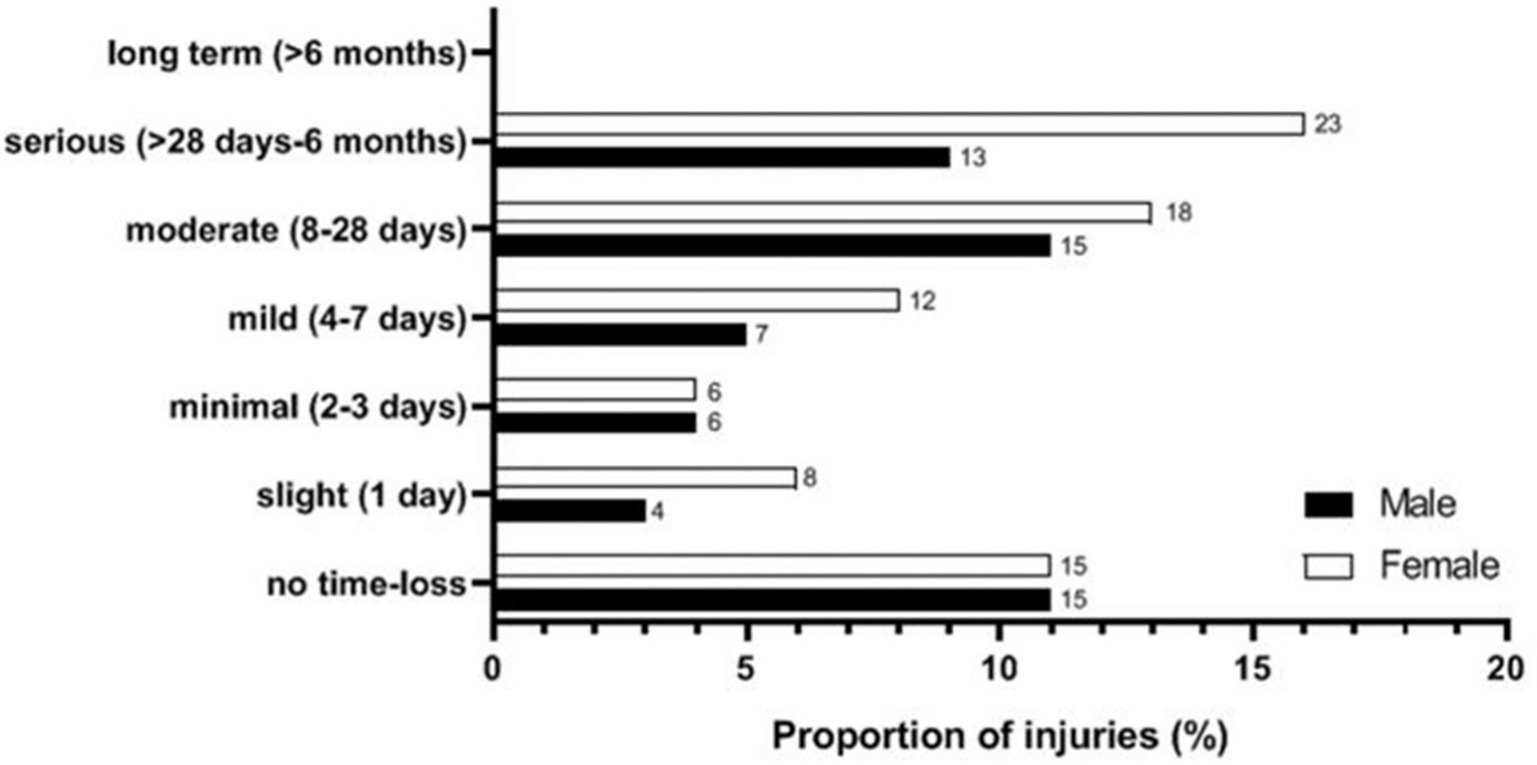
Proportion of running-related injuries (RRI) incurring time loss from distance running in days. NB, Due to rounding, not all numbers add up correctly.

Twenty-nine (59%) male participants sustained 45 RRI [IP = 59% (95% CI: 45–73); clinical incidence = 92/100 participants/year (95% CI: 82–100)] compared with 36 (56%) female participants who sustained 67 RRI [IP = 56% (95% CI: 44–68%); clinical incidence = 105/100 participants/year (95% CI: 98–111)], resulting in a risk ratio of 1.05 (95% CI: 0.77–1.45). The IR for male participants was 4.4/1,000 participation hours (95% CI: 3.3–5.9), and for female participants it was 5.4/1,000 participation hours (95% CI: 4.3–6.9), resulting in a rate ratio of 0.82 (95% CI: 0.63–1.34). Figure 1 represents the proportion of “all RRI” that resulted in time loss according to severity and sex.

#### Medical Attention RRI

Forty-four (39%) participants sustained at least one new RRI during the 12-month retrospective study period that required medical attention. These participants received medical attention for a total of 59 RRI, resulting in an IP of 39% (95% CI: 30–48), and a clinical incidence of 52/100 participants/year (95% CI: 41–63). The IR was 2.61/1,000 participation hours (95% CI: 2.59–2.63).

Fifteen (31%) male participants sustained 19 RRI [IP = 31% (95% CI: 18–44); clinical incidence = 39/100 participants/year (95% CI: 22–56)] compared with 29 (45%) female participants who sustained 40 RRI [IP = 45% (95% CI: 33–58); clinical incidence = 63/100 participants/year (95% CI: 49–76)], resulting in a risk ratio of 0.68 (95% CI: 0.41–1.11). The IR for male participants was 1.9/1,000 participation hours (95% CI: 1.84–1.90), and for female participants it was 3.2/1,000 participation hours (95% CI: 3.19–3.26), resulting in a rate ratio of 0.58 (95% CI: 0.46–1.36). Medical attention by a physiotherapist was the most common type of treatment self-reported (92%).

### Injury Characteristics

The body regions and areas of self-reported RRIs and pains or discomfort are summarised in Table 2. Excluding self-reported pains or discomfort, there were a total of 76 self-reported RRIs, representing 54% of “all RRI.” Of these 76 RRIs, 11 (14%) were classified as recurrent RRIs, while the most commonly reported mode of onset was gradual (51%) and the most commonly reported cause was overuse (84%).

**Table 2:** Self-reported RRIs and pains or discomfort by body region and area.

**Body region**	**Frequency (%)**	**IR/1,000 h (95% CI)**
**Body area**		
Lower extremity	131 (92)	5.8 (4.9–6.9)
Knee	31 (22)	1.4 (1.0–2.0)
Foot/toes	23 (16)	1.0 (0.7–1.5)
Lower leg	22 (16)	1.0 (0.6–1.5)
Thigh	17 (12)	0.8 (0.5–1.2)
Ankle	14 (10)	0.6 (0.4–1.1)
Hip	11 (8)	0.5 (0.3–0.9)
Achilles tendon	7 (5)	0.3 (0.2–0.7)
Groin	6 (4)	0.3 (0.2–0.6)
Upper extremity	1 (1)	0.1 (0.0–0.3)
Elbow	1 (1)	0.1 (0.0–0.3)
Head and trunk	10 (7)	0.4 (0.2–0.8)
Lumbar spine/lower back	8 (6)	0.4 (0.2–0.7)
Pelvis/sacrum/buttock	2 (1)	0.1 (0.0–0.4)
Total	142 (100)	6.3 (5.3–7.4)

*RRI, Running Related Injury; IR, Incidence Rate; h, hours; CI, Confidence Intervals. NB, Due to rounding, not all numbers add up correctly*.

### Training Practices

Age-group (years) based training practices and level of specialisation are presented in Table 3. ANOVA revealed a significant age-group effect for sessions per week (*p* = 0.012) with the 13–14 age-group reporting fewer sessions per week than the 17–18 age-group (*p* = 0.013). No other significant age-group effects for training practices and level of specialisation were found.

**Table 3:** Training practices and level of specialisation according to age-group (data presented as mean and SD, unless otherwise stated).

	**13–14 years (*n* = 36)**	**15–16 years (*n* = 43)**	**17–18 years (*n* = 34)**
Training Practices:			
Sessions per week^*^	4.1 (1.2)	4.9 (1.5)	5.1 (1.8)
Weeks per month	3.9 (0.2)	3.9 (0.3)	3.9 (0.2)
Months per year	11.3 (1.6)	10.9 (1.9)	10.6 (2.7)
Average session duration (min)	58.5 (20.5)	53.7 (21.0)	61.7 (16.1)
Average RPE per session (AU)	6.3 (1.3)	6.6 (1.5)	6.3 (1.3)
Average sRPE per session (AU)	379.6 (150.6)	400.8 (198.0)	393.8 (181.6)
Level of specialisation (N, %):			
Low	5 (14%)	3 (7%)	1 (3%)
Moderate	15 (42%)	14 (33%)	10 (29%)
High	16 (44%)	26 (61%)	23 (68%)

*RPE, Rating of Perceived Exertion; sRPE, Session Rating of Perceived Exertion; AU, Arbitrary Units. NB, Due to rounding, not all numbers add up correctly.*

* *p < 0.05 between groups*.

## Discussion

This study provides novel insights into RRI and training practices in a cohort of competitive adolescent distance runners. The key findings were that: (1) for “all RRI,” the IP was 68% (95% CI: 113–138), and the IR was 6.3/1,000 participation hours (95% CI: 5.3–7.4); (2) the most commonly injured body areas were the knee, foot/toes, and lower leg, with overuse being the most common cause of RRI; (3) the number of training sessions per week (i.e., frequency) increased with chronological age; and (4) a large proportion of participants (58%) self-reported a high level of specialisation.

### Running-Related Injury

For “all RRI,” the reported IP was particularly high in this population (68%), toward the higher end of data reported in adult distance runners (van Gent et al., 2007). When considering exposure time, the reported IR (6.3/1,000 participation hours), for “all RRI,” was slightly higher than that reported in two distinct cohorts of elite Swedish adolescent distance runners (4.0 to 5.3/1,000 participation hours) (von Rosen et al., 2017, 2018). The reported IR for “all RRI” was lower than that reported in a previous study on a cohort of competitive adolescent distance runners in England (25.0/1,000 participation hours) (Mann et al., 2021), alongside previous studies that have included cohorts of recreational (7.7/1,000 participation hours) and novice (17.8/1,000 participation hours) adult distance runners (Videbaek et al., 2015). Overall, these IP and IR findings suggest that the cohort of competitive adolescent distance runners in the present study maintain a greater training volume (exposure) than in other sports, whereby the higher IP may be a result of this greater training volume. That said, another likely explanation for this is that previous research has often applied a time loss and/or medical attention definition of RRI (Yamato et al., 2015), resulting in a lower IP. This is in contrast to the “all RRI” definition used within the present study.

The IR for male participants (5.9/1,000 participation hours), for “all RRI,” is higher than that reported in several other youth sports, with a similar IR to that found in youth football studies (Caine et al., 2008). A similar pattern was observed in relation to the IR for female participants (6.6/1,000 participation hours), indicating a lack of significant sex differences. This is further supported by the absence of a rate ratio between the male and female participants in this study (rate ratio = 0.89; 95% CI: 0.68–1.33). Despite these observations, these figures are notably lower than those reported in a recent study that included a similar cohort of adolescent distance runners (Mann et al., 2021). Nonetheless, comparison between these studies is challenging due to the different study designs (i.e., retrospective vs. prospective). Likewise, the studies reporting IR in a cohort of youth cross-country runners calculated this outcome according to the number of RRIs per 1,000 athletic exposures, also making comparison of results difficult (Rauh et al., 2000, 2006).

The IP for those RRIs that required medical attention was 39%, while the largest proportion of RRIs incurring time loss were categorised as “serious” (25%). Although this is an interesting finding, highlighting that a quarter of RRIs resulted in more than 28-days of time loss (up to 6-months), this may be due to recall bias. On the contrary, the large proportion of RRIs incurring “no time loss” (22%) was due to participants being able to register any physical complaint when self-reporting RRI, whereby 45% (*n* = 30) of self-reported pains or discomforts did not result in time loss.

RRIs were most commonly reported in the lower extremity, with the knee, foot/toes, and lower leg being the most frequently injured sites. These body areas are comparable to those reported in elite adult and adolescent track and field athletes (Jacobsson et al., 2012). Likewise, the most common self-reported cause of RRI was overuse, which supports previous findings (DiFiori et al., 2014). These data indicate that RRI prevention measures for adolescent distance runners should predominantly focus on reducing the risk of lower extremity RRI caused by overuse.

### Training Practices and Potential Risk Factors

The results highlight that the number of training sessions per week (frequency) are significantly different between age-groups, with a higher number of weekly sessions being recorded in the older 17–18 age-group, compared to the 13–14 age-group. This finding supports the results from a study that included a cohort of elite Australian youth track and field athletes (Huxley et al., 2014), alongside the notion that competitive athletes in “centimetres, grams, or seconds” sports increase their training during late adolescence (Moesch et al., 2011). However, no other significant differences were found between age-groups, in relation to the training practices of these athletes. This might be because a large proportion (58%) of these adolescent distance runners had a higher specialisation and broadly similar training ages, regardless of sex.

The proportion of participants (58%) self-reporting a high level of specialisation, irrespective of sex, is notably higher than that reported in previous studies across a variety of sports (Jayanthi et al., 2015; Bell et al., 2016; Post et al., 2017b). In these studies, the proportion of adolescent athletes who self-reported a high level of specialisation ranged from 28.1 to 36.4%. When accounting for the type of sport, Pasulka and colleagues demonstrated that this proportion is larger (45%) in individual sport athletes, when compared to team sport athletes (Pasulka et al., 2017). This supports the findings within the present study. As a further point, the largest proportion of participants across each age-group consistently self-reported a high level of specialisation. This is supported by the fact that both the volume and intensity of training did not differ much between age-groups, while the average number of training months per year (11.0) exceed current evidence-based recommendations (Jayanthi et al., 2013). Considering that a high level of specialisation has previously been associated with injury history in youth athletes (Post et al., 2017a), alongside the fact that distance running is an individual and late-specialisation sport (Moesch et al., 2011; Kearney and Hayes, 2018), these findings are relatively concerning. For example, a high level of specialisation may be a potential risk factor for RRI in competitive adolescent distance runners. As a result, detailed analytical epidemiology is required to explore the implications of a high level of specialisation within this population, whereby confounding factors will need to be carefully accounted for.

Lastly, it is important to recognise that the majority of participants self-reported that they had a coach (96%), included a warm-up (99%) and cool-down (98%) as part of their training sessions, and followed a strength and conditioning programme (79%). These training practices imply that injury prevention measures that include a range of neuromuscular training exercises (e.g., strength, balance, and agility) may be implementable within this specific sporting context (Emery et al., 2015). Nonetheless, further research is required to support the development of injury prevention measures for competitive adolescent distance runners.

### Methodological Limitations

The main limitations of this study included the use of a cross-sectional study design and that recruitment difficulties resulted in a limited sample size. These limitations meant that it was not possible to determine the association between RRI and potential risk factors (i.e., unable to perform logistic regression analysis). The convenience sampling method may also have led to a non-representative sample.

Recall bias is a further study limitation, whereby the accuracy of data was dependent upon self-report. This type of bias often results in participants under-reporting minor injuries, leading to an artificially greater proportion of severe injuries. While there was a high proportion of serious time loss RRI, the proportion of RRI that incurred no time loss was also elevated. Nonetheless, research has shown that participants can accurately recall the total number of injuries and injury sites when providing a 12-month self-reported history (Gabbe et al., 2003). However, as that research was based in a different sporting context, the effect of recall bias remains unclear within this study. Social desirability bias is also possible, whereby participants could have over-reported their training practices. It is also important to recognise that self-reported intensity was based on perceived exertion. Although this approach has been validated for use within this population (Mann et al., 2019), this does not mean that other physiological or biomechanical measures of intensity did not vary between participants and the different age-groups.

## Conclusions

This study found that the IR for competitive adolescent distance runners, for “all RRI,” is both slightly higher than that reported in studies that have included youth endurance athletes, and lower than the IR reported in a similar cohort of competitive adolescent distance runners and studies that solely include adult distance runners. This study also demonstrated that the IP for “all RRI” was particularly high, feasibly explained by differences in injury definition and/or the greater training volume (exposure) in competitive adolescent distance runners, when compared to other youth sports. The knee, foot/toes, and lower leg were the most commonly injured body areas, with overuse being the most common cause of RRI. The total number of training sessions per week increased with chronological age, while a large proportion of participants self-reported a high level of specialisation within this study. Due to a limited sample size, logistic regression analysis was not possible. As a result, analytical epidemiology is required to provide better insight into potential RRI risk factors.

## Data Availability Statement

The raw data supporting the conclusions of this article will be made available by the authors, without undue reservation. Requests to access the data will be considered by the authors, within the constraints of privacy and consent.

## Ethics Statement

Written parental consent and participant assent were obtained before completion of the questionnaire. Ethics approval was granted by the Sport and Health Sciences ethics committee (171206/B/02), at the University of Exeter.

## Author Contributions

RM, AB, and CW were involved in the design of the study. RM was responsible for study implementation. RM and AB did the statistical analyses. RM wrote the first draft of the manuscript, with AB making initial revisions. All other revisions by RM were circulated and commented on by AB, CW, BC, and CM. All authors read and approved the final manuscript.

## Conflict of Interest

The authors declare that the research was conducted in the absence of any commercial or financial relationships that could be construed as a potential conflict of interest.

## Abbreviations

%: Percentage
BMI: Body mass index
CI: Confidence intervals
IOC: International Olympic Committee
IP: Incidence proportion
IQR: Interquartile range
IR: Incidence rate
RPE: Rating of perceived exertion
RRI: Running-related injuries
SD: Standard deviations
sRPE: Session rating of perceived exertion
X^2^: Chi-squared test.
