# Supplementary material for: Injuries and Training Practices in Competitive Adolescent Distance Runners: A Retrospective Cross-Sectional Study
Source: Front Sports Act Living. 2021 Jun 24;3:664632. doi: 10.3389/fspor.2021.664632 (PMC8264289; doi:10.3389/fspor.2021.664632)
Supplement: Supplementary file 1 [file Data_Sheet_1.PDF]

Participant Identification: ___________________
to be completed by Prinicipal Investigator.

DISTANCE RUNNING STUDY
Online Questionnaire

SECTION #1: Demographics

**Full Name:**	**Date of Birth:** ____ / ____ / ____ Day Month Year
**Sex:** ☐ Male ☐ Female	
**Height:** ________ feet ____ inches **or** ____________ cm	**Is distance running your main sport?** ☐ Yes ☐ No*
**Weight:** ______________ stone **or** ____________ kg	**How many years have you participated in distance running?**
**Age Group:** ☐ 13-14 y ☐ 15-16 y ☐ 17-18 y	☐ 1 ☐ 2 ☐ 3 ☐ 4 ☐ 5
**Are you a member of an Athletics Club?** ☐ Yes ☐ No	☐ 6 ☐ 8 ☐ 9 ☐ 10 ☐ 11<br>☐ 12 ☐ 13 ☐ Other __________
**If you answered 'yes', which Athletics Club(s)?**	**What is your current level?** ☐ Recreational (for fun) ☐ Competitive
***If distance running is not your main sport, what is?	**How long have you participated at this level?** ________ years ________ months

SECTION #2: Performance History

**Which distance running event is your main event?** *Tick one.*<br>☐ 800 m ☐ 1,500 m ☐ 3,000 m<br>☐ 5,000m ☐ 10,000m ☐ Other<br>If 'other', which event? ____________	**Which other events do you compete in?** *Tick all that apply.*<br>☐ 800 m ☐ 1,500 m ☐ 3,000 m<br>☐ 5,000m ☐ 10,000m ☐ Other<br>If 'other', which events? ____________
**What is your personal best time for your main event?**<br>________ : ________<br>Minutes Seconds	**Do you currently have a Coach?** ☐ Yes ☐ No<br>**If 'yes', do they set your training plan?** ☐ Yes ☐ No<br>**If 'no', who sets your training plan?**
**At what level are you currently competing?** *Tick all that apply.*<br>☐ Club ☐ County ☐ Regional ☐ National ☐ International	
**What is the highest level that you have competed at?** *Tick one.*<br>☐ Club ☐ County ☐ Regional ☐ National ☐ International	

SECTION #3.1: Training Practices

How many months of the past year (12 months) did you participate in distance running?

☐ None ☐ 1 ☐ 2 ☐ 3 ☐ 4
☐ 5 ☐ 6 ☐ 7 ☐ 8
☐ 9 ☐ 10 ☐ 11 ☐ 12

How many weeks per month did you participate in distance running? *For the months where you did participate.*

☐ None ☐ 1 ☐ 2 ☐ 3 ☐ 4

How many days per week did you participate in distance running? *For the weeks where you did participate.*

☐ None ☐ 1 ☐ 2 ☐ 3 ☐ 4
☐ 5 ☐ 6 ☐ 7

How many minutes per day did you participate in distance running?

☐ None ☐ less than 1 hour ☐ 1-2 hours ☐ 3-4 hours
☐ 5-6 hours ☐ 7-8 hours ☐ Other: ____________

How long does a normal training session last for you? *Round up to the nearest hour.*

☐ less than 30 mins ☐ between 30 mins - 1 hour ☐ between 1 - 2 hours ☐ More than 2 hours

What surface do most of your training sessions take place on?

☐ Athletics Track ☐ Tarmac / Road ☐ Grass / Cross Country ☐ Other: ____________

What footwear / shoes do you wear when running on the following surfaces? *Complete table below.*

	Type of surface	Brand of shoe	Model of shoe	Duration of use (i.e. months)?
**1**	Athletics Track			
**2**	Tarmac / Road			
**3**	Grass / Cross Country			
**4**	Other: ______________			

Do you include a warm-up as part of your training sessions? ☐ Yes ☐ No

In 'yes', please describe your typical warm-up: ________________________________

Do you include a cool-down as part of your training sessions? ☐ Yes ☐ No

In 'yes', please describe your typical cool-up: ________________________________

Do you do any strength and conditioning (physical preparation), in addition to your distance running? ☐ Yes ☐ No

In 'yes', please describe what this typically involves and who sets this training: ____________________

How many times did you compete in distance running events in the past year (12 months)?

☐ None ☐ Less than 10 ☐ Between 10 and 20 ☐ Other: ____________

SECTION #3.2: Training Diary

Thinking about the last seven days of your distance running training (i.e. a typical week), please complete the following training diary. When entering session duration and distance covered, please estimate as best as possible. For session intensity, please choose a number on the CR-10 Borg Scale (on back of questionnaire), by answering the following question: 'how hard was your workout?'.

Day of week	Session number	Total session duration (min)	Total distance covered (km)	Type of training session	Session intensity	Specific details about the training session
Example	1	*45*	*7*	*6 x 800 m (2 min recovery)*	*7 = very hard*	On an atheltics track, in spikes. Started the session at 9am
	2	*30*	*6*	*30 minute easy run*	*2 = easy*	*On tarmac, in regular trainers.* Started the session at 6pm.
Monday	1					
	2					
Tuesday	1					
	2					
Wednesday	1					
	2					
Thursday	1					
	2					
Friday	1					
	2					
Saturday	1					
	2					
Sunday	1					
	2					

SECTION #4: Athletic Identity Measurement Scale

For each statement, please circle one number from 1 (strongly disagree) to 7 (strongly agree) that best represents your answer.

	Strongly Disagree						Strongly Agree
I consider myself an athlete.	1	2	3	4	5	6	7
I have many goals related to sport.	1	2	3	4	5	6	7
Most of my friends are athletes.	1	2	3	4	5	6	7
Sport is the most important part of my life.	1	2	3	4	5	6	7
I spend more time thinking about sport than anything else.	1	2	3	4	5	6	7
I need to participate in sport to feel good about myself.	1	2	3	4	5	6	7
Other people see me mainly as an athlete.	1	2	3	4	5	6	7
I feel bad about myself when I do poorly in sport.	1	2	3	4	5	6	7
Sport is the only important thing in my life.	1	2	3	4	5	6	7
I would be very depressed if I were injured and could not compete in sport.	1	2	3	4	5	6	7

SECTION #5: Injury and Medical History

In the past year (12 months), have you had an injury that resulted from distance running participation, irrespective of the need for medical attention or time loss from the sport? This includes training and conditioning for distance running.

☐ Yes ☐ No If 'yes', please list in table below:

Injury Date	Injury Type	Session Type	Surface	Body Part	Time loss	Treatment (if any)	How were you injured?
i.e. month and year	*Sprain, bruise, etc.*	*Intervals, race, etc.*	*Track, grass, etc.*	*Left elbow, ankle, etc.*	*1 day, 3 weeks, etc.*	*None, first aid, Doctor, physio, massage, etc.*	*fall, repetitive overuse, strenuous move, etc.*

Were any of these injuries the same injury happening again? This can include injuries beyond the previous 12 months.

☐ Yes ☐ No If 'yes', please list (#'s): ______________________________

Do you have any injuries that are ongoing?

☐ Yes ☐ No If 'yes', please describe these injuries: ______________________________

Are you currently receiving treatment for any of these injuries?

☐ Yes ☐ No If 'yes', please describe these injuries: ______________________________

Participant Identification: ___________________

to be completed by Prinicipal Investigator.

Are you currently taking medication for any of your injuries?

☐ Yes ☐ No If 'yes', please list medication: ☐ Paracetemol
☐ Ibuprofen
☐ Other: ____________

Do you currently take any medication on a regular basis?

☐ Yes ☐ No If 'yes', please list medication: ☐ Paracetemol
☐ Ibuprofen
☐ Asthma Inhaler
☐ Other: ____________

Are you currently taking any supplements for performance and/or health reasons (vitamins, minerals, protein powder, etc.)?

☐ Yes ☐ No If 'yes', please list supplements, the dose and how long you have been *taking the supplement:* ______________________________

____

____

Have you ever been diagnosed by a physician with a bone fracture, arthiritis, and/or other muscle or bone related condition?

☐ Yes ☐ No If 'yes', please describe (include year): ____________________

Have you had surgery in the past year?

☐ Yes ☐ No If 'yes', please describe: ______________________________

In the past year (12 months), have you had any pain, discomfort, or physical problems during running that you did not list as an injury?

☐ Yes ☐ No If 'yes', please list in the table below.

**Date**	**Session Type**	**Surface**	**Body Part**	**Time loss**	**Treatment (if any)**	**Description**
i.e. month and year	*Intervals, race, etc.*	*Track, grass, etc.*	*Left elbow, ankle, etc.*	*1 day, none, 3 weeks, etc.*	*None, first aid, Doctor, physio, massage, etc.*	*Provide short overview*

SECTION #6: Specialisation

N.B. These questions were combined in Section #1 when applied as a baseline questionnaire (Chapter 7).

**Is distance running more important to you than any other sport?**	☐ Yes	☐ No
**Have you quit other sports in order to focus on distance running?**	☐ Yes	☐ No
**Do you train or participate in distance running for more than 8 months of the year?**	☐ Yes	☐ No

In the past year, how many months, weeks and hours per week (on average) did you participate in a school PE class?

________ number of months ________ number of weeks ________ hours per week

Based on the past year, did you participate in any sports on a weekly basis (NOT including PE class or running)?

☐ Yes ☐ No If 'yes', please estimate the average number of hours per week you participated in each sport:

SPORT	hrs/wk, mo/yr	SPORT	hrs/wk, mo/yr	SPORT	hrs/wk, mo/yr
Aerobics	,	Floor hockey	,	Speed skating	,
Athletics	,	Football	,	Swimming	,
Badminton	,	Golf	,	Tennis	,
Baseball	,	Gymnastics	,	Ultimate Frisbee	,
Basketball	,	Hiking/ Scrambling	,	Triathlon	,
Boxing (incl. kick)	,	Horse riding	,	Volleyball	,
Canoeing	,	Kayaking	,	Waterpolo	,
Caving	,	Lacrosse	,	Weight training	,
Climbing	,	Martial arts	,	Wrestling	,
Cycling - Mountain	,	Rafting	,	*Other:	,
Cycling - Road	,	Rugby	,		,
Dance	,	Skateboarding	,		,
Dirt biking	,	Skiing - Downhill/Alpine	,		,
Diving	,	Skiing - Cross-country	,	*Please describe other:*	
Field hockey	,	Snowboarding	,		
Figure skating	,	Squash	,		

Section #8: Further Research

**Are you be willing to take part in any further research studies?**	☐ Yes	☐ No
**Are you be willing to take part in any further research studies?**	☐ Yes	☐ No

Thank you for completing this questionnaire

**If yes, please provide us with the full name of your parent or guardian, in addition to their contact details:**<br><br>**Full Name:** ______________________ **Email:** ____________ **Telephone:** ____________
